# Recent advances in the chemical recycling of polyesters

**DOI:** 10.1016/j.fmre.2024.05.014

**Published:** 2024-06-04

**Authors:** Sheng Wang, Jing Li, Xiaohong Li, Yingfeng Tu

**Affiliations:** a Jiangsu Key Laboratory of Advanced Functional Polymer Design and Application, State and Local Joint Engineering Laboratory for Novel Functional Polymeric Materials, College of Chemistry, Chemical Engineering and Materials Science, Soochow University, Suzhou 215123, China; b Key Laboratory of Ionic Rare Resources and Environment of Ministry of Natural Resources, Jiangxi College of Applied Technology, Ganzhou 341000, China

**Keywords:** Polyesters, Chemical recycling, Depolymerization, Cyclic monomers, Catalysis, Circular polymer economy

## Abstract

Whilst polymers have played a significant role in the development of modern society, a rapid growth of polymer waste is disadvantageously influencing communities and ecosystems across the world. Constructing a closed-loop life cycle of polymer materials is urgently in demand. Chemical recycling of polyesters, which can be recovered to the pristine monomers or transformed to other value-added products, has been considered as an appealing recycling approach to circular polymer economy and attracted enormous attention in the last several years. This review highlights some recent progress in the chemical recycling of diverse polyesters, including commercialized poly(lactic acid), poly(*ε*-caprolactone), poly(ethylene terephthalate), as well as various novel chemically recyclable polyesters and polycarbonates. Eventually, based on these technological developments, we discuss the remaining challenges and identify promising research opportunities, providing insights into future directions for achieving a genuine closed-loop polymer economy.

## Graphical abstract

**Figure:**
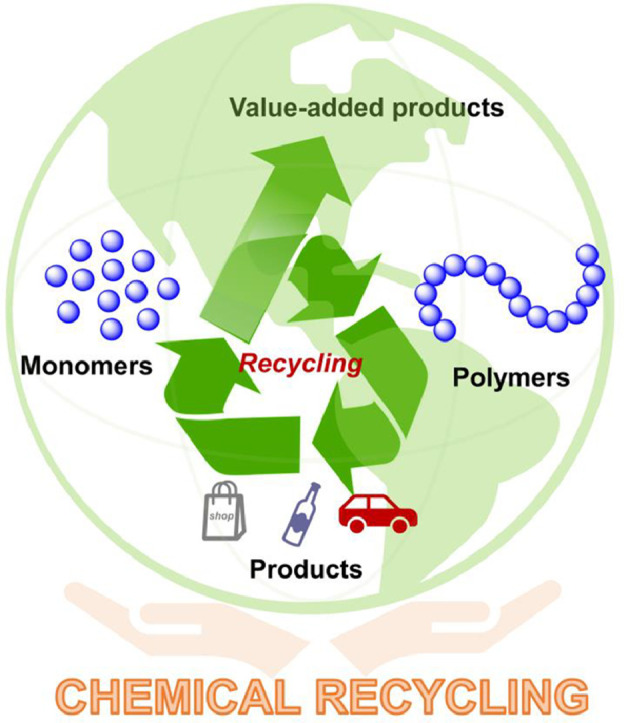

## 1 Introduction

Due to the advantages of cheapness, light weight, and excellent performance, synthetic polymers have fueled modern economies and become a necessity in modern life. Nevertheless, large quantities of polymer wastes have been thrown away and accumulated in the environment, due to the massive industrial manufacture and ineffective recycling, causing a serious threat to the human society and ecosystem [1,2]. The main treatment methods of polymer wastes include incineration and landfill [3,4]. However, these wastes are often mismanaged and brought into oceans. Only a small portion of polymer wastes has been successfully recycled through physical or chemical treatment. To address the serious polymer pollution, exploiting novel methodologies to effectively recover commodity polymeric materials, or designing new high-performance recoverable polymers becomes quite urgent.

The main recycling methods of polymeric materials can be divided into mechanical recovery, energy recovery, as well as chemical recycling [5,6]. Mechanical recovery is the traditional pathway for plastic recycling; however, inevitable chain scission or degradation during melt reprocessing tends to significantly degrade the mechanical performance of the obtained plastic products [6]. For energy recovery, polymer materials are burned to generate heat or power, which is suitable for complex systems, whereas there are emissions of carbon dioxide and toxic pollutants. Thereby, it also belongs to a low value-added recovery technology. By comparison, chemical recycling is able to recover the initial precursor monomers via depolymerization or creatively transform polymer waste to other value-added materials. When the recovered monomers are reutilized to fabricate the pristine polymer materials, their structure and performance can be well maintained without apparent degradation. Chemical recycling can efficiently change the conventional “linear material economy mode” to “circular material economy mode”, and supply a significant opportunity to overcome the end-of-use problem of polymer materials [5]. Thereby, chemical recycling is identified as a promising recycling approach to construct a circular polymer economy.

For polyolefins, the sp^3^-hybridized C—H and C—C bonds are inherently inert and possess very good stability with activation barriers *E*_a_ up to 150∼300 kJ mol^−1^, resulting in high depolymerization temperature (commonly > 400 °C), which makes the recovery of these polymers difficult. By contrast, polyesters linked via many polar sp^2^-hybridized C=O bonds are recognized as ideal candidates for chemical recycling due to the susceptibility of ester carbonyl bonds to nucleophilic attack [5]. On the other hand, polyesters are playing an increasingly important role in our daily life with quite rapid development rate. Thus, most progress in catalytic chemical recycling pertains to polyester-based plastics.

A variety of excellent review articles have been published on the chemical recycling of polyesters [[5], [6], [7], [8], [9]]. Most of these review articles are focused on the chemical recycling of polymer materials based on the equilibrium between ring-opening polymerization and ring-closing depolymerization. For commercial diol/diacid-type semiaromatic and aliphatic polyesters, their chemical recycling methods are not involved in these review articles. With the rapid development of this field, many breakthroughs have been achieved in the last few years. To fill this gap, this review summarizes the recent progress in the chemical recycling of not only polyesters derived from small cyclic monomers but also diverse diol/diacid-type polyesters, especially the work published in the last five years. Moreover, cyclodepolymerization is an important strategy to achieve the chemical recycling of many commercial diol/diacid-type semiaromatic and aliphatic polyesters, as the resultant cyclic oligoesters could be utilized to synthesize an assortment of high value-added polyester-based materials like multiblock copolyesters [10]. This method has not been systematically introduced in recent reviews, and is thus also summarized in the present review. Considering the progress of chemically recyclable polythioesters has been summarized in a recent review [11], here we will not introduce it again.

This review is divided into two sections: (1) chemical recycling of polyesters derived from small cyclic monomers (with large ring strain) including lactones or lactides, cyclic ether-esters, carbonates, and depsipeptides; (2) chemical recycling of diol/diacid-type polyesters including semiaromatic polyesters like poly(alkylene terephthalate)s (PATs) and poly(ethylene 2,5-furandicarboxylate) (PEF), as well as various diol/diacid-type aliphatic polyesters. Based on these technological developments, the remaining challenges and promising research opportunities are eventually discussed to offer our insights into the future directions for genuine closed-loop polymer economy.

## 2 Chemical recycling of polyesters derived from small cyclic monomers

For small cyclic monomers, their polymerization processes are commonly enthalpy favorable and entropy unfavorable, namely Δ*H* < 0, Δ*S* < 0. According to the formula Δ*G* = Δ*H* − *T*Δ*S*, there is a ceiling temperature (*T*_c_) at which Δ*G* is equal to zero and polymerization-depolymerization is under equilibrium. At *T* < *T*_c_, the polymerization is favorable, while depolymerization is favored at *T* > *T*_c_. Polymer composition has significant influences on *T*_c_. For polyesters derived from small cyclic monomers, the polymerization-depolymerization equilibrium can be easily shifted towards the formation of initial monomers at high temperature above *T*_c_. The presence of suitable catalysts is capable of not only improving the depolymerization selectivity but making the depolymerization conditions milder. This kind of polyesters have unique chemical recyclability and have attracted enormous research interests in the last few years. In this section, we focus on the important progress mainly in the past five years.

### 2.1 Chemical recycling of polyesters derived from cyclic lactones or lactides

Aliphatic polyesters have been viewed as a kind of significant biocompatible and biodegradable polymers, which can be easily accessed via various ring opening polymerization (ROP) methods from cyclic esters like lactones or lactides. In this section, we will first introduce the latest progress in chemical recovery of the commercial aliphatic polyesters derived from cyclic monomers like PLLA and PCL. Then many breakthroughs in other novel aliphatic polyesters such as poly(*γ*-butyrolactone), poly(*δ*-valerolactone), as well as their analogues, have also been introduced.

#### 2.1.1 Chemical recycling of PLLA

**Fig. 1:**
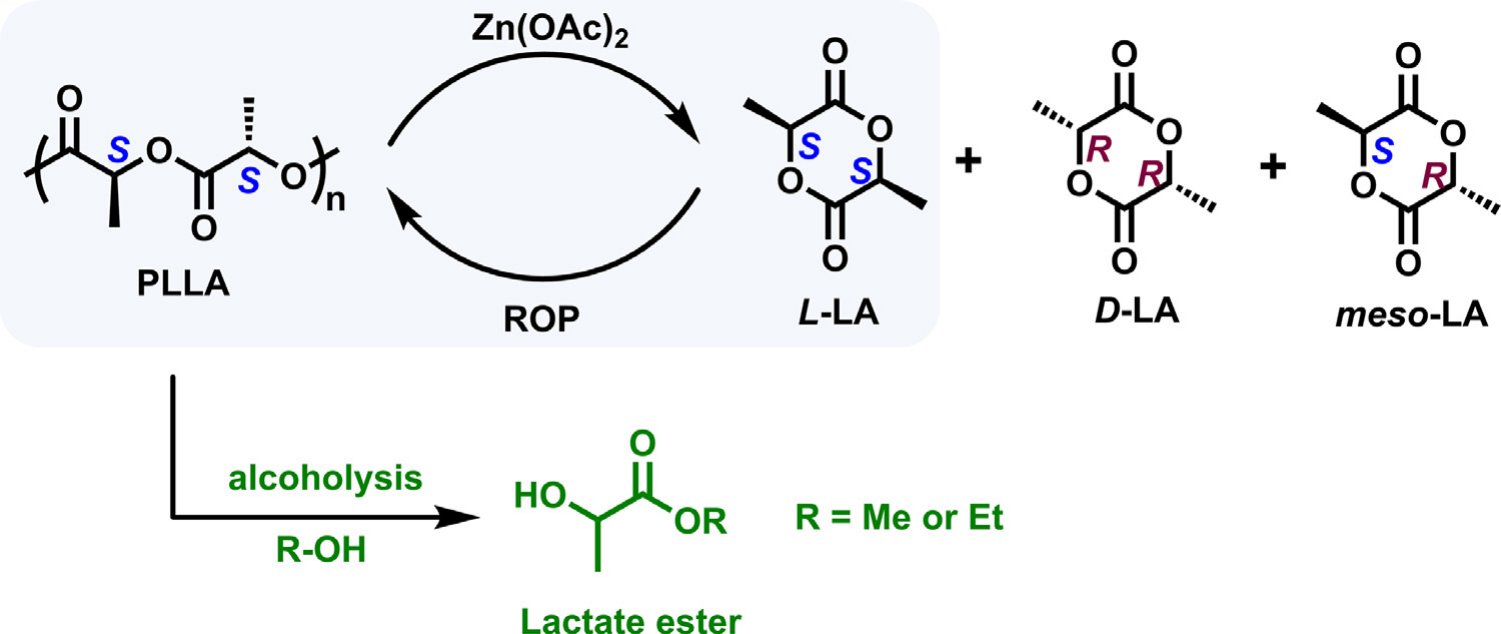
**Polymerization and depolymerization of PLLA**.

Poly(*L*-lactic acid) (PLLA) is the most productive aliphatic polyester among bio-based plastics. The repeating lactic acid monomer can be fabricated using biorenewable resources like corns and sugar. Industrially, PLLA is produced via melt ROP of *l*-lactide (*L*-LA) without usage of any solvents. Due to the intrinsic biocompatibility, excellent biodegradability and mechanical properties, PLLA has been widely applied in the food packaging and biomedical industries [5]. However, due to its slow degradation rate under landfilling conditions or in sea conditions, PLLA waste also can become a nonnegligible plastic pollution contributor if inappropriately handled at its end-of-life. Chemical recycling of PLLA waste to recover highly pure *L*-LA is still a pivotal challenge. During thermal degradation at high temperatures, a variety of degradation products, including LA diastereomers (*D*-, *L*-, and *meso*-LA) [5,12], cyclic oligomers, acrylic acid, acetaldehyde, and CO_2_, will be generated (Fig. 1). Complicated purification procedures are needed to afford *L*-LA with high purity before ROP, otherwise the existence of *meso*-lactide structure would lead to low crystallinity and poor mechanical performance of reproduced PLA.

In 2020, Enthaler et al. [13] reported the depolymerization of PLLA waste to fabricate *L*-LA with Zn(OAc)_2_ as catalyst at high temperature (200–210 °C) and high vacuum (6 mbar) conditions, in which the yield and selectivity could reach 98% and 88%, respectively. Moreover, this catalysis system still exhibited an excellent selectivity towards PLLA depolymerization even with the interference of other plastics like PCL and Nylon 6. Nonetheless, it is still a formidable task to further increase the selectivity of the chemical recycle process.

Hydrolysis of PLLA to produce lactic acid is an important recycling method. Nevertheless, high temperatures (120 ∼ 350 °C), high pressure up to > 10 bar, and concentrated base or acid catalysts like NaOH, Ca(OH)_2_, and H_2_SO_4_ are commonly required [14]. In 2014, Liu et al. used ionic liquid 1-butyl-3-methylimidazolium acetate ([Bmim][OAc]) as catalyst for PLA hydrolysis in a relatively mild condition [15]. Calcium lactate with 76% yield was attained at around 130 °C within 2 h. Recently, Yu et al. demonstrated the use of a cheap organocatalyst diphenyl phosphate (DPP) for efficient hydrolysis of PLA into lactic acid or oligolactic acid [16]. The hydrolysis was carried out at 160 °C in the presence of small amount of water without the need of organic solvents or high pressure. The C=O groups in PLA could be easily activated by the ionized hydrogen ions of DPP, while its phosphoryl oxygen interacted with water, which enhanced its nucleophilicity, enabling a fast PLA hydrolysis. Moreover, the oligomers containing DPP catalyst could be reutilized to produce high-quality PLA, thereby realizing the closed-loop recycling. Additionally, when mixed with other common packaging plastics like polyethylene (PE), polypropylene (PP), and PET, DPP-catalyzed PLA hydrolysis was not compromised, showing a good selectivity.

By alcoholysis, PLLA also can be selectively converted to value-added lactate esters, which not only can be utilized as a green solvent but the premonomer for preparing *L*-LA [17,18]. Therefore, PLLA alcoholysis is considered as a very promising recycling strategy. An assortment of catalysts, such as Zn(OAc)_2_, Sn(Oct)_2_, 4-dimethylaminopyridine (DMAP), and 1,5,7-triazabicyclo[4.4.0]dec-5-ene (TBD), can be used to achieve this strategy [5]. In 2011, Sanchéz and Collinson [19] used Zn(OAc)_2_ as catalyst to selectively degraded PLLA into methyl lactate (Me-LA) from the PLLA/PET mixtures with Me-LA yield of 65%. In 2020, Enthaler et al. [20] found that Sn(Oct)_2_ is capable of favoring the methanolysis of PLLA to achieve Me-LA in high yields even at low catalyst loadings. Moreover, Enthaler and coworkers also reported the efficient catalytic activity of organic base DMAP for PLLA methanolysis under microwave irradiation [21]. Remarkably, high Me-LA yield up to > 99% was achieved at 180 °C in a short reaction time (approximately 10 min). Besides, Leibfarth and coworkers demonstrated TBD to be a highly effective catalyst for PLLA alcoholysis [22]. Even at room temperature, over 90% ethyl lactate (Et-LA) yield could be attained within 3 min, which was able to be further broadened to an assortment of alcohols like BnOH, BuOH and MeOH. Recently, McKeown and coworkers demonstrated that cheap tetramethylammonium methyl carbonate could act as an efficient organocatalyst to achieve PLLA methanolysis at 50 °C [23], in which the Me-LA yield was close to 100% within 1 h depolymerization time.

#### 2.1.2 Chemical recycling of PCL

**Fig. 2:**
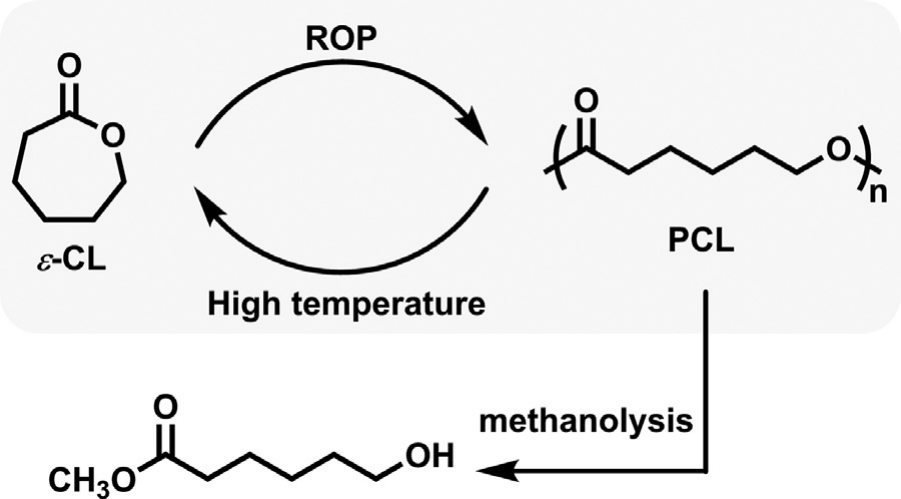
**Polymerization and depolymerization of PCL**.

Poly(*ε*-caprolactone) (PCL) is another important commercial biodegradable polyester. It is generally fabricated by ROP using *ε*-caprolactone (*ε*-CL) as monomers and has been widely utilized as packaging, biomedical, and pharmaceutical materials, owing to its good biocompatibility and excellent mechanical properties. Extensive attention has also been drawn on the heat-induced degradation of PCL to recover *ε*-CL (Fig. 2). Commonly, only when the degradation temperature is raised to ∼430 °C, does the PCL depolymerization to *ε*-CL take place, following an unzipping mechanism [5]. The thermal degradation products are complex, including oligomers, *ε*-CL, 5-hexenoic acid, water, and CO_2_. The usage of catalyst can effectively reduce the temperature of depolymerization. For instance, by using Bu_2_Sn(OMe)_2_ as catalyst, the depolymerization process is able to undergo successfully at 260 °C under high vacuum condition (0.01 mbar) [24], affording *ε*-CL in a high yield up to 82%.

Very recently, Byers et al. [25] demonstrated a simple ZnCl_2_-poly(ethylene glycol) (PEG600) catalyst system that was able to efficiently depolymerize PCL back to *ε*-CL in high yields (up to 98%) under reactive distillation conditions. A random chain scission mechanism was proposed, in which the active catalyst species possess one equivalent of ZnCl_2_ per ethylene glycol repeating unit in PEG600. Their recycling strategy is also suitable to selectively depolymerize other polyesters like PLLA, poly(*δ*-valerolactone), poly(*ω*-pentadecalactone) as well as various polycarbonates back to corresponding constitute monomers. When mixed with other plastic wastes such as PET, linear low-density PE, and PP, selective PCL depolymerization was convinced to be feasible. On the other hand, PCL methanolysis is also an interesting approach to producing value-added methyl 6-hydroxyhexanoate product, which has been successfully realized under a mild condition by Xu and Wang et al. in 2020 [26], using Zn(HMDS)_2_ as degradation catalyst.

#### 2.1.3 Chemical recycling of poly(butyrolactone) derivatives

**Fig. 3:**
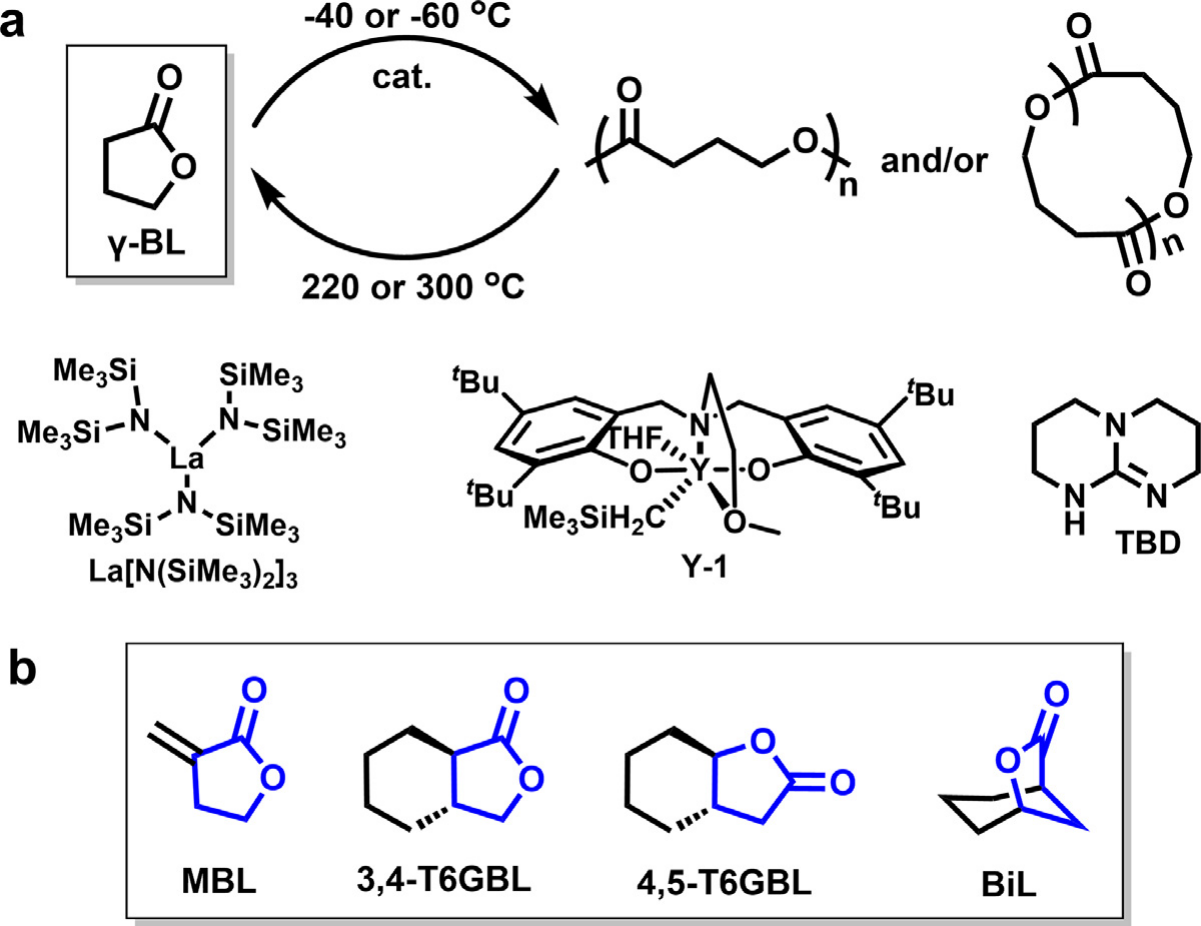
(a) Reversible polymerization/depolymerization cycles of poly(γ-butyrolactone). (b) Chemical structures of other *γ*-BL analogues.

Derived from biorenewable succinic acid, *γ*-butyrolactone (*γ*-BL) shows immense potentials in synthesizing novel homo- or co-polyesters. However, *γ*-BL had been regarded as “nonpolymerizable” before 2016, attributed to the reason that five-membered ring possesses quite low strain energies. To surmount the detrimental thermodynamics during the γ-BL ROP, Chen et al. [27] put forward a novel ROP strategy to fabricate poly(γ-butyrolactone) (P*γ*BL) at a quite low temperature (−40 °C) and a high monomer concentration by using rare earth catalysts like yttrium complexes (Y-1) or La[N(SiMe_3_)_2_]_3_, and molecular weight of resultant linear or cyclic polyesters is up to 30.2 kg mol^−1^ (Fig. 3a). These significant breakthroughs opened an avenue to PγBL synthesis. Notably, P*γ*BL displayed complete recoverability and could be selectively transformed back into the pristine monomer through melt depolymerization at 220 °C (linear polymer) or 300 °C (cyclic polymer) in less than 60 min. Besides, the rapid depolymerization of P*γ*BL can be achieved via chemolysis at ambient temperature under the catalysis of TBD or La[N(SiMe_3_)_2_]_3_.

Since then, an assortment of elegant catalysts have consecutively been developed for *γ*-BL ROP, including cyclic trimeric phosphazene base (CTPB) [28], CTPB/urea [29], alkoxides/urea [30], and *^t^*BuP_4_ [31] and *^t^*BuP_4_/ thioureas [32]. Besides, a series of diverse *γ*-BL analogues such like *α*-methylene-*γ*-butyrolactone (MBL) [33,34], *trans*-six-membered ring-fused-*γ*-BL [35,36], and bridged 6-oxabicyclo[3.2.1]octan-7-one [37] (Fig. 3b), also have been demonstrated to be polymerizable and the corresponding polyesters are depolymerizable by using metal or organic base catalysts.

#### 2.1.4 Chemical recycling of poly(valerolactone) derivatives

**Fig. 4:**
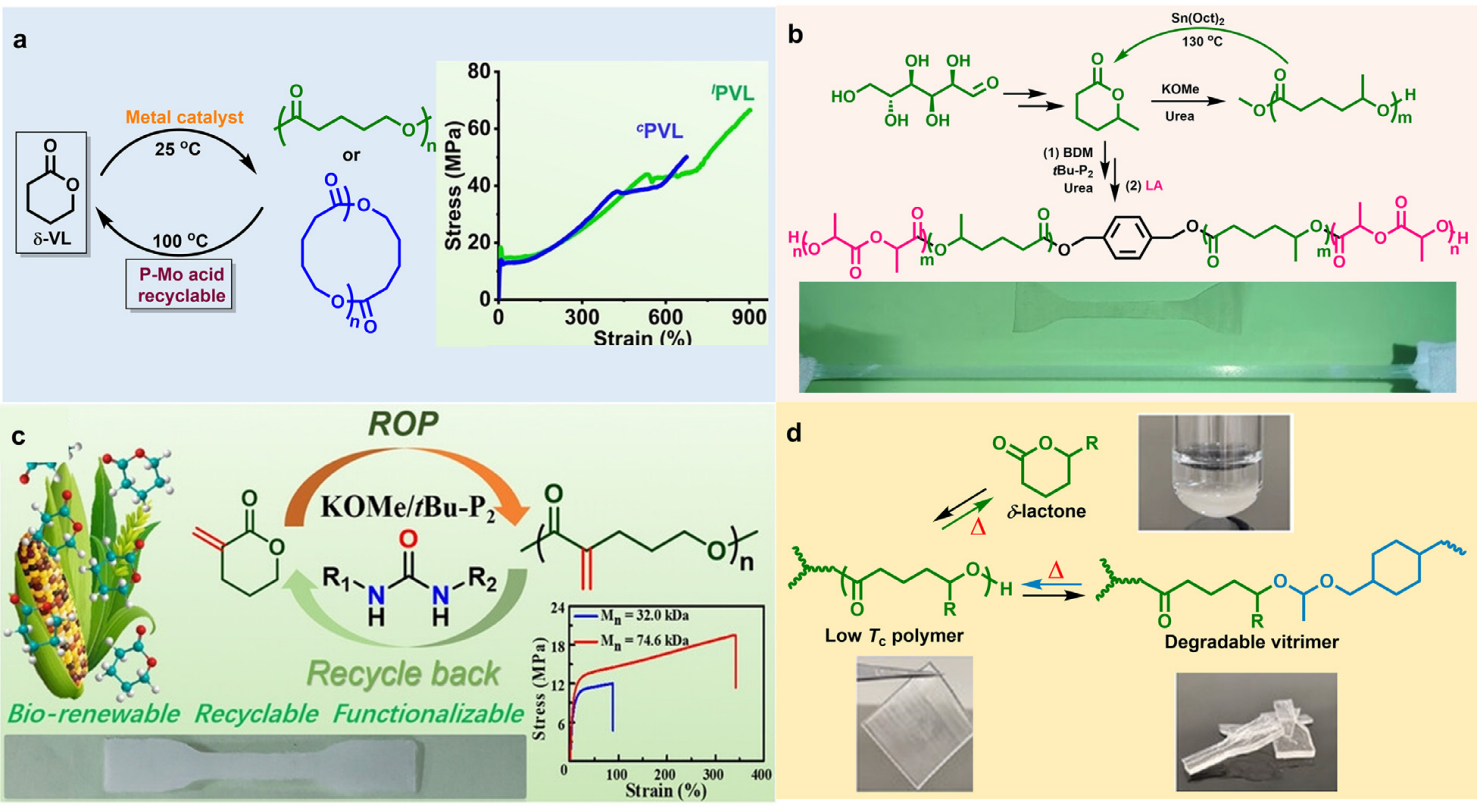
**Polymerization and depolymerization of an assortment of poly(valerolactone) derivatives**. Reproduced from Ref. [[40], [41], [42], [43]] with permission from Wiley-VCH Verlag GmbH.

Apart from commercial PLA, the polymerization and depolymerization behaviors of many other six-membered cyclolactone monomers also have attracted considerable attention in the last few years, such as *δ*-valerolactone (*δ*-VL) derivatives [38,39] and *δ*-caprolactone (*δ*-CL). In contrast to five-membered *γ*-BL, six-membered *δ*-VL has greater ring strain, making its ROP relatively more thermodynamically favorable. The most reported poly(*δ*-valerolactone) (PVL) materials suffer from relatively low *M*_n_ values below its approximated critical entanglement molecular weight, resulting in poor mechanical performance. Recently, Xu and Chen et al. demonstrated an effective ROP of *δ-*VL using metal (Y, La, and Zn) complex catalysts to achieve high molecular weight PVL (*M*_n_ ∼ 66 kg/mol) [40], which displays excellent mechanical properties with a high tensile strength (*σ*_B_) ∼ 66.6 MPa and tractility (*ε*_B_) ∼ 904%, as well as toughness of approximately 308 MJ m^−3^ (Fig. 4a). Moreover, quantitative degradation of PVL to highly pure *δ-*VL can be achieved at 100 °C with a commercial phosphomolybdic acid as catalyst [40]. Remarkably, the recovered *δ-*VL monomer can be directly polymerized to regenerate similar PVL, demonstrating a circular production process, and the phosphomolybdic acid catalyst also can be recycled enabling repeated depolymerization with little decrease of its catalytic efficiency.

On the other hand, the ROP of biosourced *δ*-CL has recently been studied using a variety of organic base or acid catalysts. In 2022, Li et al. achieved the controlled ROP of *δ*-CL by biurea/*^t^*Bu-P_2_ binary catalysts (Fig. 4b) [41]. The resulting P*δ*CL is capable of chemical recycling to near-quantitatively generate pristine *δ*-CL by Sn(Oct)_2_-catalyzed thermolysis. As shown in Fig. 4c, polyesters bearing functional vinyl groups are able to be produced from bio-sourced bifunctional *α*-methylene-*δ*-valerolactone (*α*-MVL) by ROP catalyzed using biurea/^t^Bu-P_2_ catalyst system [42]. The resultant polyester behaves as a tough plastic and can be thermally degraded to the pristine *α*-MVL monomer in a high yield (up to ∼96%). The depolymerization process took place through an unzipping pathway from ω terminal hydroxy group following a zero-order kinetics. Very recently, Qi et al. reported a depolymerizable poly(*δ*-lactone) vitrimer bearing dynamic acetal linkages for both reprocessability and monomer recovering purpose (Fig. 4d) [43]. They further demonstrated that this high-performance poly(*δ*-lactone) vitrimer is able to potentially serve as a sustainable cross-linked polymer material to replace the traditional soft elastomers.

### 2.2 Chemical recycling of polyesters derived from cyclic ether-ester monomers

**Fig. 5:**
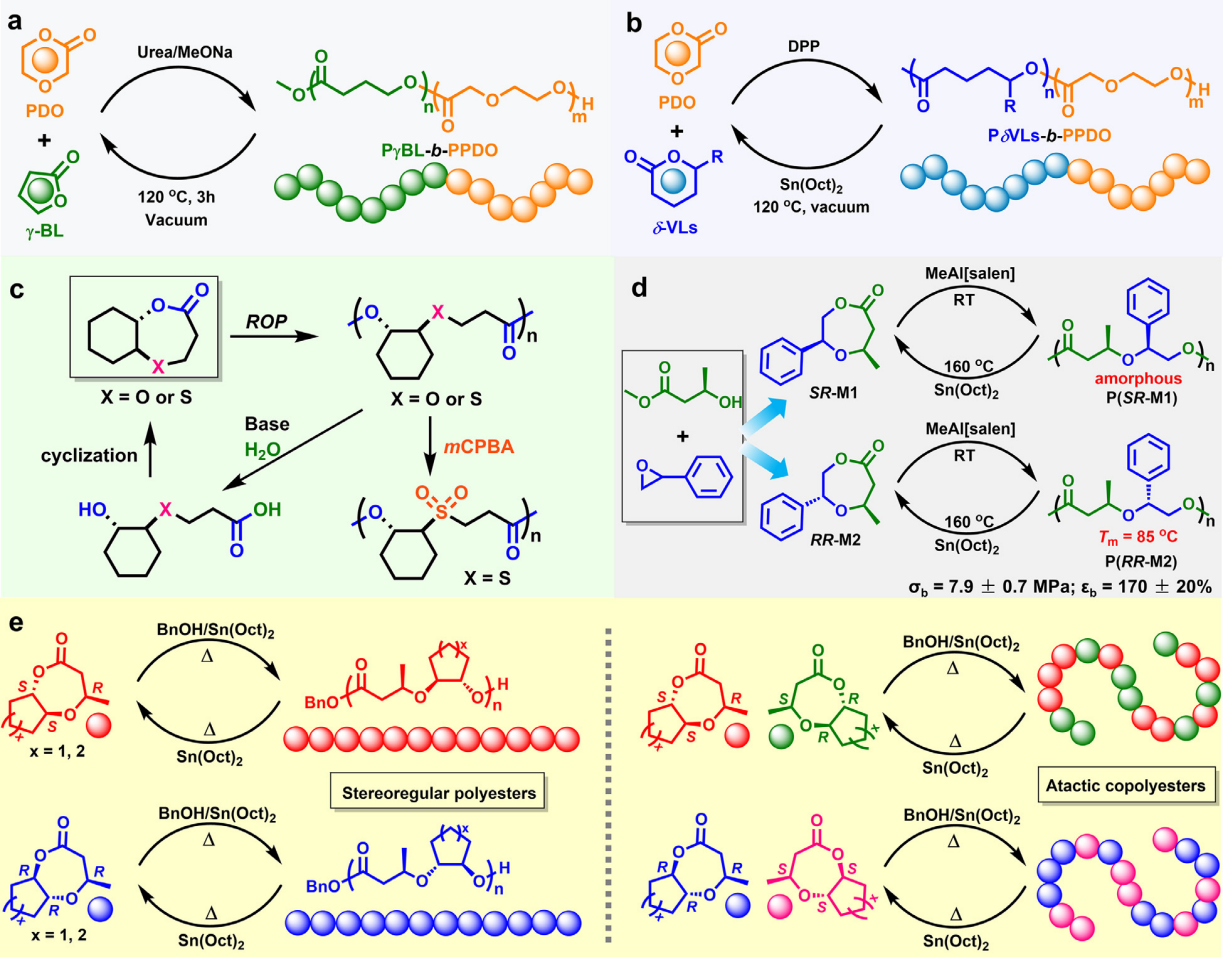
Polymerization and depolymerization of PDO with γ-BL (a) or *n*-alkyl substituted *δ-*VLs (b), bicyclic monomers OTO and DXO based on 1,4-oxathiepan-7-one and 1,5-dioxepan-2-one (c), chiral 7-methyl-2-phenyl-1,4-dioxepan-5-one (d), and bicyclic ether-ester monomers containing *trans*-fused cyclopentyl or cyclohexyl moieties (e).

Polyesters derived from ether-ester monomers are also important chemically recyclable polymers. In 2022, Wang and Wu et al. successfully used ureas/alkoxides catalytic system to fabricate diblock copolymers, poly-(*γ*-butyrolactone)-*block*-poly(*p*-dioxanone) (PγBL-*b*-PPDO), through a sequential copolymerization of six-membered *p*-dioxanone (PDO) and five-membered γ-BL in one pot [44]. By simple low-temperature (120 °C) pyrolysis under vacuum, the resulting copolyesters present outstanding degradability for efficient conversion into the initial PDO and γ-BL monomers with a high yield up to > 95% (Fig. 5a). By virtue of their discrimination in physicochemical properties, PDO and γ-BL can be separated selectively with a high isolation purity over 99%. Their repolymerization is realized to attain PγBL-*b*-PPDO copolymers with almost the same chemical components and thermal stability, proving the chemically closed-loop recycling concept. Subsequently, chemically recyclable diblock copolymers, poly(*n*-alkyl-valerolactones)-*block*-poly(*p*-dioxanone) (PVLs-*b*-PPDO) [45], were also fabricated from an assortment of biorenewable *n*-alkyl substituted *δ-*VLs and PDO as monomers via similar sequential copolymerization (Fig. 5b).

In 2022, Zhu and coworkers used the fused-ring idea to synthesize two novel bicyclic monomers OTO and DXO [46], derived from 1,4-oxathiepan-7-one and 1,5-dioxepan-2-one (Fig. 5c). These two monomers can undergo living ROP with Zn complex catalyst to yield highly stereoselective polyesters with well-tailored *M*_n_ and narrow polydispersity. The introduced sulfur atoms and *trans*-ring fusion structure in cyclic ester monomers can apparently increase the resultant polyesters’ crystalline behavior and thermostability. By immersing in LiOH aqueous solution, these polymers are able to be hydrolyzed completely to the corresponding hydroxyl acid precursor, which can be further reconverted to the polymerizable monomers via lactonization reactions.

Shen and Li et al. successfully converted the methanolysis products of poly(3-hydroxybutyrate) to fabricate a couple of O-heterocyclic lactone enantiomers bearing phenyl pendent [47], which could further undergo ROP to prepare chemically recyclable poly(ether-ester)s (Fig. 5d). The monomers’ stereo-configuration had a great influence on the thermodynamics and kinetics of ROP, as well as the mechanical and thermal performance of resulting poly(ether-ester)s. In comparison with the amorphous P(*SR*-M1), P(*RR*-M2) was a semi-crystalline polyester (X_c_ ∼ 34%) with *T*_m_ ≈ 85 °C, and could behave like a tough plastic, the tensile strength and elongation at break of which was up to 7.9 MPa and 170%, respectively. Moreover, the resulting polyesters are able to be recovered back to the pristine cyclic monomers in the yield of 85% through Sn(Oct)_2_-catalyzed solution depolymerization at 160 °C, indicating a reversible recycling process. By contrast, the depolymerization yield was reduced to about 22% in P(*SR*-M1) with ω-terminal acrylate, evincing an unzipping pathway and the existence of transesterification during depolymerization process.

Recently, Li and Shen et al. also fabricated a series of bicyclic ether-ester type enantiopure aliphatic monomers containing *trans*-fused cyclopentyl or cyclohexyl moieties (Fig. 5e) [48], which can undergo stereoretention ROP using catalyst Sn(Oct)_2_ to afford stereoregular chiral polyesters. By comparison with the amorphous atactic polymers produced from racemic isomers, the attained stereoregular polyesters present higher crystallinity with the relatively higher melting temperature. Notably, these high-performance polyesters are also capable of depolymerizing back to the pristine enantiopure cyclic monomers in both solution and bulk under the catalysis of ZnCl_2_ or Sn(Oct)_2_, evincing a perfect “monomer-polymer-monomer” recycling process.

**Fig. 6:**
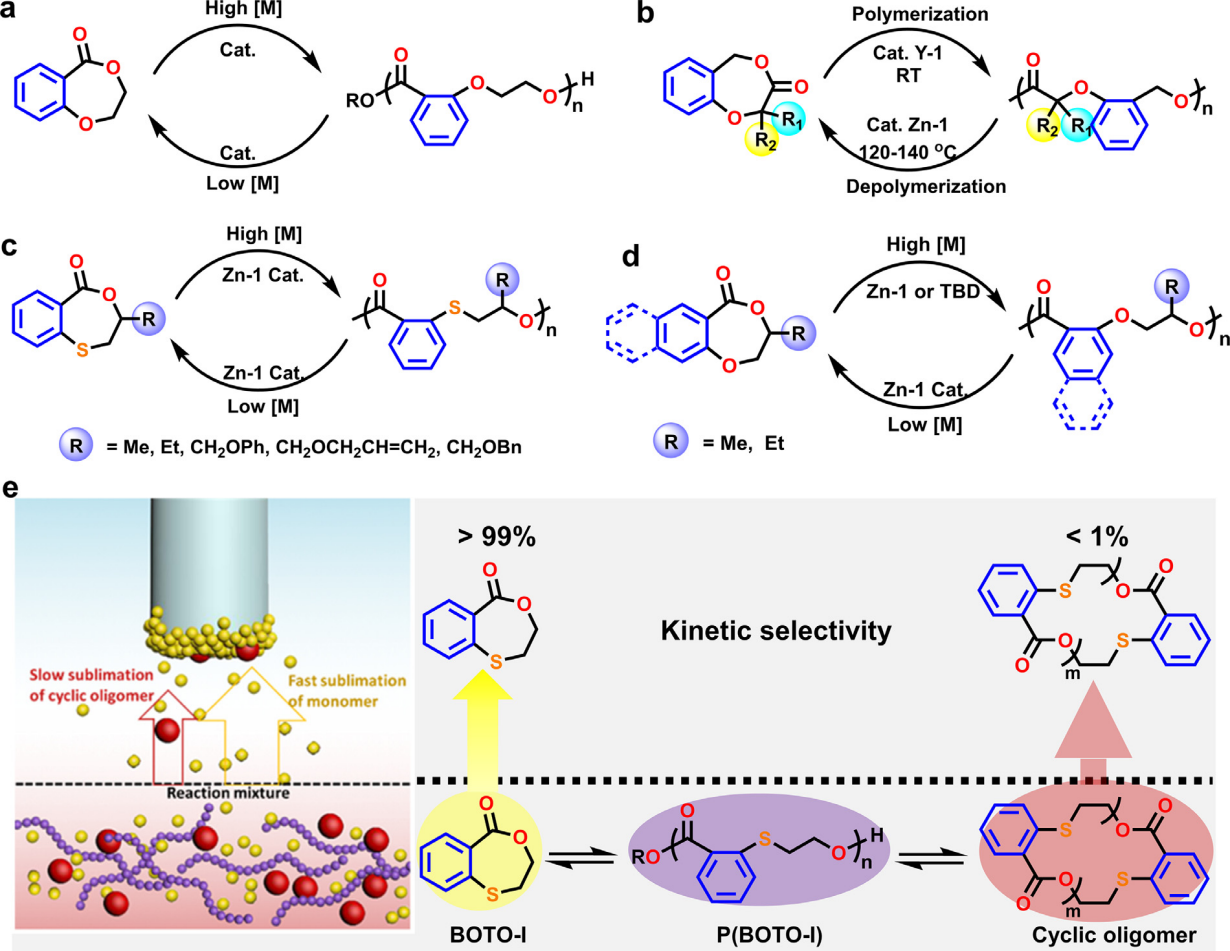
**A variety of aromatic-aliphatic poly(ether ester)s featuring reversible polymerization–depolymerization cycles**. (e) Reproduced from Ref. [54] with permission from American Chemical Society.

The above polyesters commonly possess aliphatic main-chain backbone, showing relatively low thermal behavior. Incorporating aromatic groups into polyester backbones has been verified as an effective strategy to improve the thermal and mechanical performance of the resultant materials, due to the enhanced rigidity of aromatic ring structures. However, most of aromatic polyesters presented the inferior chemical recyclability and poor degradability. In 2016, Shaver et al. [49] successfully synthesized poly(2-(2-hydroxyethoxy)benzoate) (P2HEB) with aluminium Salen or organocatalysts from commercially available 2,3-dihydro-5H-1,4-benzodioxepin-5-one (2,3-DHB) monomer. This aromatic-aliphatic polyester displays a glass-transition temperature (*T*_g_) of approximately 27 °C and good crystallization capacity during cooling process. Impressively, the same MeAl[Salen] catalyst was also able to catalyze a remarkably selective depolymerization back to the 2,3-DHB monomer at low concentrations (Fig. 6a) [50]. Moreover, the P2HEB block in P(2HEB-*b*-3HB) copolymers can be selectively degraded with only the P3HB block remaining, evincing the potential application of degradable monomers in the adjustment of macromolecular structures.

In 2021, Zhu and coworkers synthesized a variety of novel bio-sourced seven-membered cyclic esters bearing aliphatic and aromatic moieties (Fig. 6b) [51], which could be converted to the corresponding semiaromatic polyesters with a high *M*_n_ ∼ 438 kg/mol through alkyl yttrium catalyzed ROP. The resulting polymers can form stereocomplex exhibiting significantly increased *T*_m_ (up to 175 °C), and possess a tunable *T*_g_ (1 ∼ 79 °C) by altering the substituent group. Remarkably, in bulk or solution, all the obtained polyesters can be easily depolymerized into the initial monomers, convincing the establishment of a circular life cycle. Soon after, they utilized epoxides and thiosalicylic acid to synthesize a type of novel aromatic cyclic esters (Fig. 6c) [52], which can further undergo ROP to produce high-molecular-weight aromatic polyesters with narrow polydispersities. By forming stereocomplex, polyesters showed high thermostability, good crystallinity (*T*_m_ ∼ 209 °C), and polyolefin-like outstanding mechanical behaviors. Notably, these polyesters are able to selectively transform back to corresponding monomers with excellent yields (89%–98%) in dilute solution. This also works when the more rigid naphthalene groups are introduced into the polyester backbone to apparently enhance its thermal and mechanical properties (Fig. 6d) [53], with excellent chemical recyclability.

Inspired by Shaver's work, Li et al. [54] also synthesized three benzo-thia-caprolactones with structures similar to P2HEB (Fig. 6e). DSC results indicate that the obtained polyesters are semi-crystalline polymers displaying slow melt crystallization rate. Remarkably, all three BOTO monomers and their corresponding polyesters displayed excellent polymerization-depolymerization characteristics. In solution, catalytic depolymerization is very fast with poor selectivity, generating the mixtures of monomers and oligomers. By comparison, the melt depolymerization in bulk can selectively and efficiently give highly pure BOTO monomers.

### 2.3 Chemical recycling of polycarbonates

Polycarbonate (PC) is a kind of polymer embedding a lot of carbonate groups in the polymer chain [55], generally categorized into aliphatic and aromatic PCs. Among them, the aromatic PCs possess very superior mechanical properties, mainly including bisphenol A type PCs, which are important engineering plastics. By contrast, aliphatic polycarbonates (APCs) have received considerable attention and are widely applied in medical device materials and packaging applications, because of their potential degradability, good biocompatibility, and non-toxicity.

**Fig. 7:**
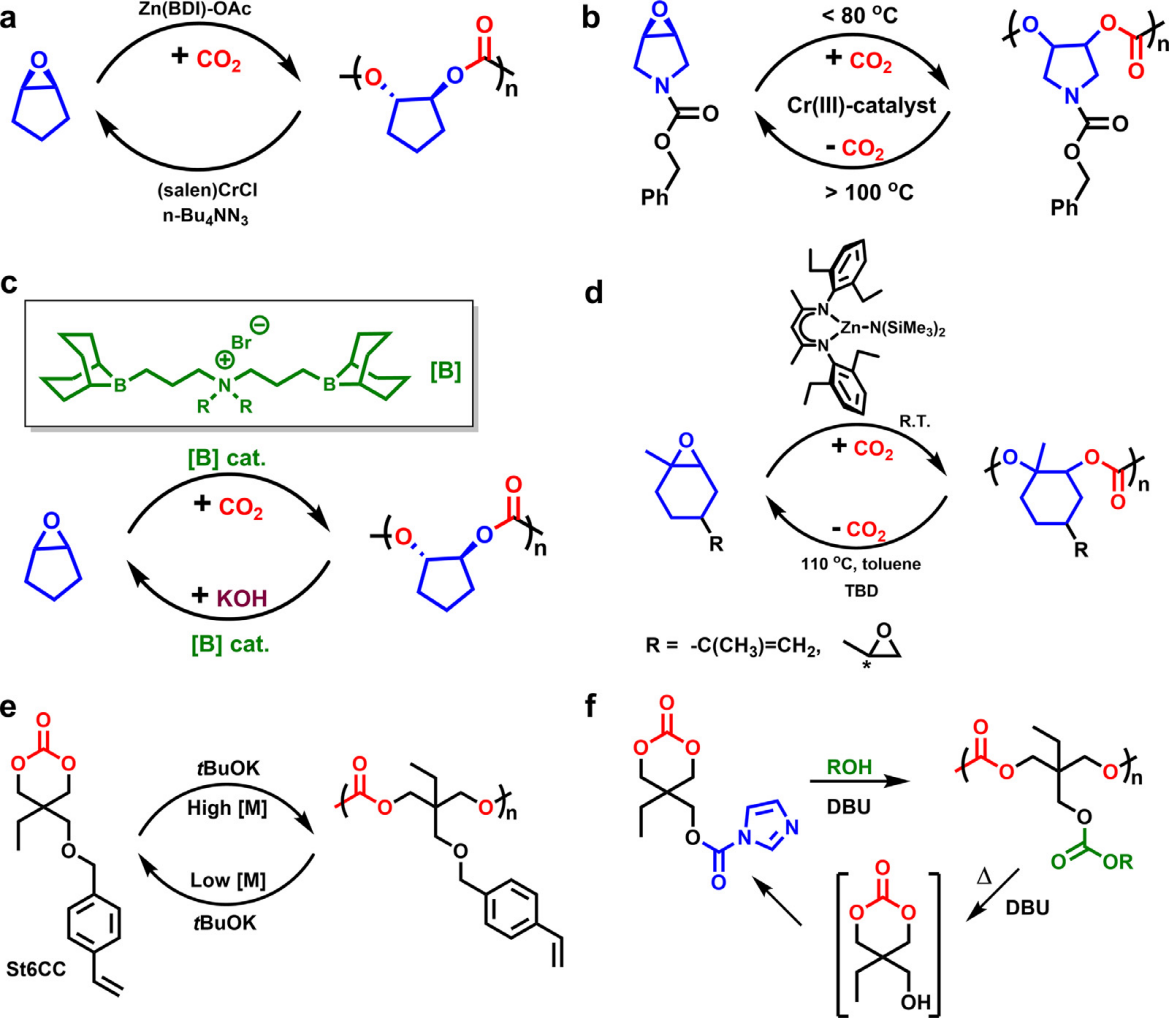
**An assortment of aliphatic polycarbonates showing reversible polymerization–depolymerization cycles**.

Recently, enormous attention has been paid to understanding APCs’ depolymerization behavior and recyclability. In this field, Darensbourg is one of the most important pioneers and has conducted systematical investigations on the depolymerization of diverse APCs prepared from epoxides and CO_2_. Their results substantiated that the depolymerization process of APCs at modest temperatures underwent endwise scission reaction to selectively produce five-member-ring carbonates (Fig. 7a) [[56], [57], [58]]. They demonstrated that the degradation process involved the APCs’ unzipping from the chain terminal through a backbiting mode. Moreover, they also carried out high-accuracy CBSQB3(+) calculations [57], for achieving the energy barriers for some APCs to conduct alkoxide backbiting to produce CO_2_ and corresponding epoxide monomers. For most polycarbonates, free energy barriers to generate epoxide (12.7–17.4 kcal mol^−1^) are higher than that to cyclic carbonate formation (10.7–14.6 kcal mol^−1^). But for poly(cyclopentene carbonate), the formation of epoxide exhibits a relatively lower energy barrier than that of forming cyclic carbonates (CCs), which clearly explained why the base-catalyzed depolymerization products of poly(cyclopentene carbonate) is 1,2-epoxycyclopentane, while other APCs depolymerize to their respective CCs.

In 2017, Lu and coworkers [59] fabricated a type of recyclable and nonpetroleum-based polycarbonate from the copolymerization of CO_2_ and furfural-derived N-heterocyclic epoxide, in which over 99% carbonate units are successfully depolymerized back into the epoxide precursors at > 100 °C (Fig. 7b). Noticeably, the switch between depolymerization and copolymerization processes could be readily achieved via the reversible variation of external temperature for at least three cycles without the obvious generation of byproducts. Recently, this group also demonstrated five more fully recyclable PCs [60], derived from 1-alkoxylcarbonyl-3,4-epoxy pyrrolidine epoxides. Thanks to the introduction of alkyl chains, APCs’ thermostability and crystalline ability were apparently enhanced. Furthermore, the existence of stable five-membered pyrrolidine ring is capable of ensuring the outstanding recyclability with high quantitative conversions under mild degradation conditions. No obvious byproducts such as diol or carbonate were observed.

Moreover, Wu et al. [61] reported metal-free poly(cyclopentene carbonate) by copolymerizing CO_2_ and cyclopentene oxide, which is also capable of recovering back to the epoxide precursors in a near-quantitative yield (> 99%) using organoboron catalysts via a random chain scission pathway combined with chain unzipping (Fig. 7c).

The limonene oxide (LO) produced from the bio-sourced limonene, can selectively copolymerize with CO_2_ in the existence of catalysts to afford degradable bio-based APCs. In 2017, Rieger et al. [62] reported the dynamic equilibrium between depolymerization and copolymerization at the critical concentrations of LO at a moderate temperature. In the same year, Sablong et al. [63] used the strong organic base TBD to catalyze the depolymerization of poly(limonene carbonate) (PLC). They demonstrated that TBD was able to efficiently deprotonate the PLC with OH terminals, thereby resulting in rapid quantitative degradation into the corresponding initial LO monomers at 110 °C through backbiting reaction mode (Fig. 7d). Their results evince that this fully bio-based PLC has excellent recoverability.

Apart from the ring-opening copolymerization of epoxy alkanes/CO_2_, APCs can be synthesized via ROP of CCs. The anionic polymerization of six-membered CCs bearing diverse substituted groups at the 5-position presents dynamic equilibrium character, suggesting that these monomers can be utilized to fabricate chemically recyclable PCs. Commonly, high monomer concentrations favor the ROP process to achieve polycarbonates, which in turn are capable of depolymerizing back to corresponding precursor CCs in dilute solution. However, these PCs were unable to be fully recycled. In 2005, Endo and coworkers reported the ROP of several six-membered CCs like 2-allyloxymethyl-2-ethyl-trimethylene carbonate and 5-ethyl-5-[(*p*-vinylphenyl) methoxymethyl]-1,3-dioxan-2-one (St6CC) [64], which displayed dynamic equilibrium polymerization character in the presence of *t*BuOK. For instance, St6CC was able to undergo ROP catalyzed by *t*BuOK to attain poly(St6CC) in 80% yield with *M*_n_ ∼ 14 kg mol^−1^ (Fig. 7e), which went back to the starting monomer at a dilute concentration in 60% yield. Recently, Reineke et al. [65] developed a novel one-pot tandem functionalization/ROP of a CC with imidazolecarboxylate group to fabricate PCs bearing functional pendants (Fig. 7f). The obtained PCs exhibited a good recoverability for CC monomer bearing a OH pendant, which was able to undergo further refunctionalization with imidazolecarboxylate, thereby building up a circular polymer economy. Since the APCs obtained by ROP of six-membered CCs are unable to completely recover back to the starting cyclic monomers, great efforts are still required to be devoted in the future to improve the recyclability of six-membered CCs.

The above polyesters or polycarbonates are mainly derived from small cyclic monomers, and most of them possess the polymerization-depolymerization equilibrium controlled by thermodynamics. Above *T*_c_, the depolymerization process can occur in the presence of suitable catalysts, and commonly starts from the reactive terminated hydroxyl groups by backbiting reaction in a chain unzipping mode. The depolymerization conditions of some typical polyesters derived from small cyclic monomers are summarized in Table 1. Either dilute solution or bulk thermal depolymerization can be applied to achieve the chemical recycling of polyesters. For aliphatic polyesters, the pristine cyclic monomers are capable of recovering in high yield and purity using bulk thermal depolymerization based on sublimation or distillation. By contrast, when aromatic groups were introduced in the mainchain or side chain of polyesters, its chemical recycling was mainly carried out in dilute solutions, due to the high boiling points of corresponding cyclic monomers.

**Table 1:** **Depolymerization conditions of some typical polyesters**^a^**derived from small cyclic monomers**.

Structure of cyclic monomers	Catalysts	Conditions	Yields	Ref.
	Zn(OAc)_2_ (0.4 mol%)	200–210 °C, 6 mbar	98%	[13]
	Bu_2_Sn(OMe)_2_ (0.5 mol%)	260 °C, 0.01 mbar	82%	[24]
	ZnCl_2_ (10 wt%)/PEG600	160 °C	98%	[25]
	No catalyst	220 °C or 300 °C	100%	[27]
	TBD or La[N(SiMe_3_)_2_]_3_ (2.0 mol%)	in CH_2_Cl_2_ or THF at 25 °C	100%	
	Phosphomolybdic acid (2.0 wt%)	100 °C	98%	[40]
	Sn(Oct)_2_ (0.5 wt%)	130 °C, distillation under reduced pressure	99%	[41]
	Sn(Oct)_2_ (0.5 wt%)	130 °C, 200 Pa	96%	[42]
	No catalyst	200 °C, distillation under reduced pressure	100%	[43]
	Sn(Oct)_2_ (1.0 wt%)	120 °C, 80 Pa	>96.4%	[44,45]
	Sn(Oct)_2_ (10 mol%)	160 °C in 1,3,5-trimethylbenzene	85%	[47]
	Sn(Oct)_2_ (5.0 mol%)	160 °C in 1,3,5-trimethylbenzene	87%	[48]
	ZnCl_2_ (5.0 mol%)	180 °C, 200 Pa	80%	
	MeAl[salen] (1.4 mol%)	60 °C, *c* = 0.17 M	>90%	[49]
	Sn(Oct)_2_ (2.5 mol%)	200 °C in a sublimation device	>93%	[54]
	Zn-1 catalyst (2.0 mol%)	120–140 °C, in toluene *c* = 0.02 M	>78%	[51]
	*t*-BuOK (5.0 mol%)	20 °C, in THF *c* = 0.1 M	60%	[64]

a These polyesters could be transferred to the pristine cyclic monomers during depolymerization.

## 3 Chemical recycling of diol/diacid-type polyesters

### 3.1 Chemical recycling of poly(alkylene terephthalate)s

Poly(alkylene terephthalate)s (PATs) are a type of polyesters bearing a repeating aromatic unit linked to an aliphatic alkyl chain with ester bonds, and have dominated the market for decades. Variation in the alkyl chain length has a significant impact on the thermomechanical performance of PATs. Well-known examples of PATs include poly(ethylene terephthalate) (PET), poly(trimethylene terephthalate) (PTT), and poly(butylene terephthalate) (PBT), the alkyl chain of which contains two, three or four methylene groups, respectively. Among them, PET occupies the most prominent member and is an important semicrystalline polyester, which can be fabricated by condensation polymerization of dimethyl terephthalate (DMT) or terephthalic acid (TPA) with ethylene glycol (EG), or by bis(2-hydroxyethyl) terephthalate (BHET) [14]. By far, PET's production is the largest among all polyesters with an annual production exceeding 80 million tons, and has been widely applied in numerous economic fields due to its excellent strain resistance and low permeability. The massive usage of diverse PET materials has brought about a tremendous cost to our ecological environment. The chemical recycling of PET waste is urgently in demand.

**Fig. 8:**
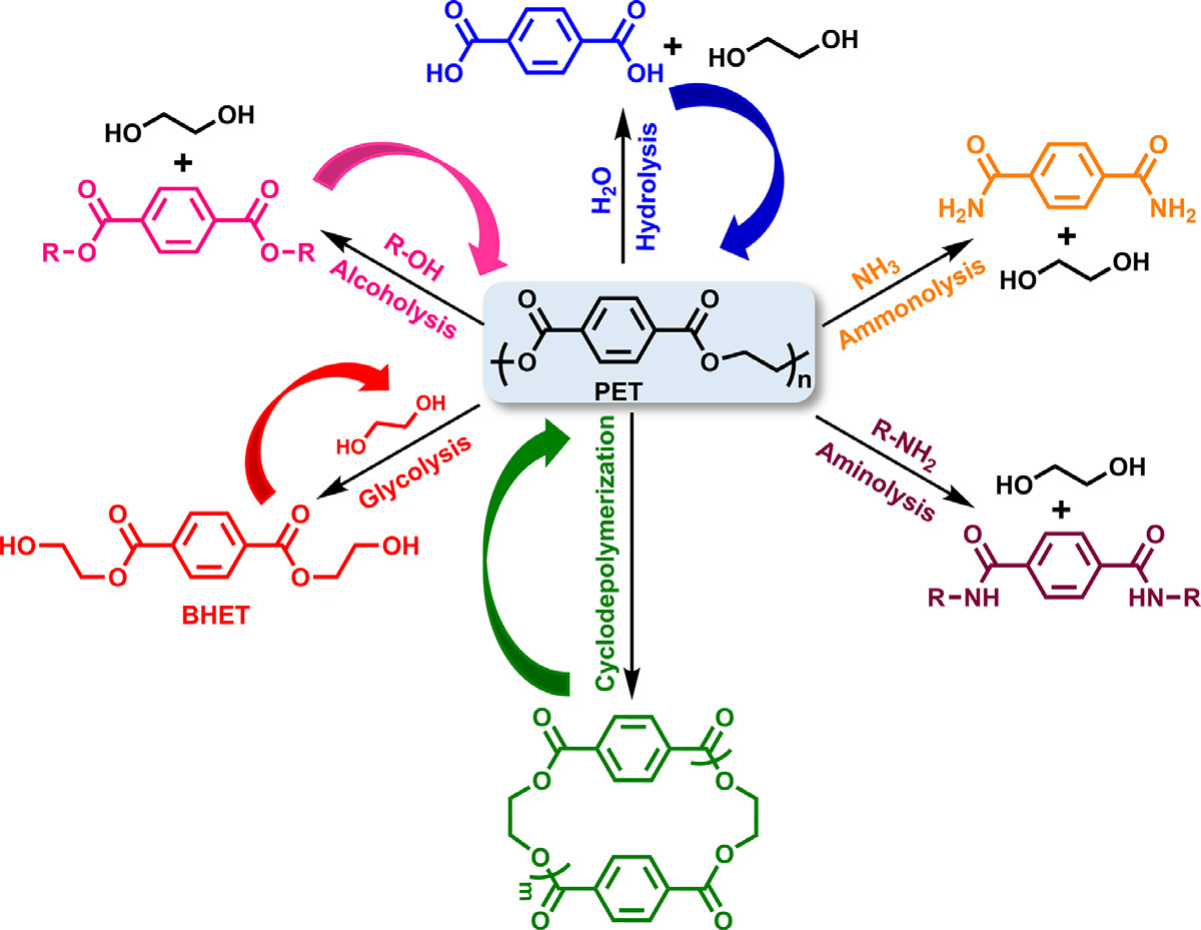
**Diverse depolymerization/upcycling strategies for PET chemical recycling**.

In chemical recycling, PET can be either totally or partially degradated, generating various monomers such as BHET, DMT, TPA, EG, linear or cyclic oligomers, and other chemicals. An assortment of depolymerization strategies have been exploited such like hydrolysis, alcoholysis, glycolysis, ammonolysis, aminolysis, and cyclodepolymerization according to the used chemical agents (Fig. 8). These recycled small products can be repolymerized to yield PET or converted to various value-added polymer materials. Generally, these recycling methods are also suitable for other PATs like PTT and PBT, due to their similar chemical structures. Here, we mainly highlighted the most recent progress in PATs’ chemical recovery, especially PET.

#### 3.1.1 PAT hydrolysis

PAT hydrolysis is based on the hydrolysis of PAT ester groups with water at high temperature and pressure in ether acid, alkaline, or neutral media, mainly yielding TPA and alkylene glycols as final products. Typically, concentrated H_2_SO_4_, HNO_3_, H_3_PO_4_, *etc.* can be used for acid hydrolysis of PATs, while alkaline hydrolysis relies on NaOH or KOH solution at a high concentration up to > 4.0 wt% [14]. Moreover, neutral hydrolysis can be achieved using alkali metal acetate as transesterification catalyst like. However, PAT hydrolysis usually brings about many critical issues such as the high corrosiveness of equipment and disposal of large quantities of strongly basic or acidic waste water. Besides, the generated TPA's purity is usually not high, which commonly requires further complex purification processes resulting in the increment of cost. Thus, it is difficult for PAT hydrolysis to be widely applied in the scale-up industrial production for the food-grade recycled PAT products.

In 2020, Cha and coworkers introduced microwave radiation into PET hydrolysis using ZSM-5 as the acidic catalyst in neutral media [66]. The TPA yield is close to 100% within 1 h reacting time. The hydroxonium ions generated by Brønsted acid catalyst H^+^@ZSM-5 could convert PET's C=O groups into OH groups forming carbo-cation, which favored the nucleophilic attack of water and reduction of activation energy. Remarkably, the H^+^@ZSM-5 catalyst displayed excellent recoverability, which can maintain a good catalytic activity even after several cycles. Afterwards, Thielemans *at al.* [67] developed an energy-saving non-aqueous hydrolysis in anhydrous alcohol by ingenious combination of KOH and microwave irradiation. The microwave-assisted heating can permit a complete conversion (100%) of PET into TPA at 120 °C in a very short time about 1 min.

Very recently, Cochran et al. demonstrated a novel energy-efficient chemical recovering of PET in water [68], by subtly introducing the diethyl 2,5-dihydroxyterephthalate (DHTE) segment into PET copolymers, in which the participation of neighboring group was able to selectively autocatalyze the hydrolysis at DHTE sites. With the increment of DHTE addition, the degradation rate constant was monotonically raised, whereas the corresponding thermal activation barrier was obviously reduced. The major degradation products are EG, TPA, bis(2-hydroxyethyl) terephthalate dimer and 2,5-dihydroxyterephthalic acid, which can be utilized to reproduce the prime polyesters.

Inspired by hydrolases [69], Niu et al. recently developed a binuclear complex featuring biomimetic Zn‒Zn sites, which was capable of catalyzing PET hydrolysis. A high activity of 577 g_PET_ d^−1^ g_catal_^−1^ was achieved at 90  °C and pH 13 for scalable PET recycling [70]. The recovered TPA had > 99% purity and was able to be used directly for the preparation of bottle-grade PET. Moreover, this binuclear catalyst also displayed consistent performance towards the mixtures of post-consumer PET with diverse common plastics like PLA, PCL, PE, and polystyrene (PS), indicative of an excellent depolymerization selectivity.

#### 3.1.2 PAT alcoholysis

The alcoholysis of PATs is conducted under high temperature and pressure conditions to afford alkyl terephthalate and alkylene glycols. Zinc acetate is the most widely used metal acetate to catalyze transesterification, while other metal acetate, aryl sulfonic acid salts, *etc*. have also been verified to show good catalytic activity in transesterification [71]. Recently, green solvents like ionic liquids (ILs) and deep eutectic solvents (DES) also exhibited noticeable catalytic activity in some cases of PAT alcoholysis.

In 2013, Liu et al. reported the PET alcoholysis using *n*-butanol under the catalysis of a Brönsted-Lewis acidic IL [HO_3_S-(CH_2_)_3_-NEt_3_]Cl-ZnCl_2_ to produce dibutyl terephthalate [72], the yield of which is up to 95.3% at 205 °C for 8 h. Notably, this IL catalyst exhibits good reusability and its catalytic effectiveness does not present significant decline after seven recycles. Later, Hu and Zhang et al. successfully achieved the 2-ethyl-1-hexanol alcoholysis of PET with choline chloride-based DES (Zn(OAc)_2_/ChCl) as alcoholysis catalyst [73]. Alcoholysis reactions proceed well at temperatures between 155 and 185 °C, and the yield of dioctyl terephthalate can reach 84.7% under the optimized reaction condition (ChCl/Zn(OAc)_2_ 1:1, 180 °C, 1 h). The Zn^2+^ of IL catalyst could attack PET's C=O groups to produce carbon cation following acid-catalyzed mechanism, while the COOH^−^ was able to attack the OH group of 2-ethyl-1-hexanol and activate C=O groups simultaneously, obeying base-catalyzed mechanism. Thereby, the rate of PET alcoholysis was highly accelerated based on this synergistic effect.

Despite the utilization of diverse alcohols, more attention has been paid towards methanolysis in the past several years. Under the catalysis of Zn(OAc)_2_ or other transesterification catalysts, PET methanolysis commonly required very high pressures over 20 atm and high temperatures above 180 °C to attain DMT and EG [14], which is capable of repolymerization for recycled PET. Albeit the good tolerance of methanolysis process towards a variety of pollutants, the existence of water will seriously obstruct PAT methanolysis and poison the catalysts to generate an assortment of undesirable azeotropes. At an early stage, Sako and coworkers studied the feasibility of PET depolymerization in supercritical CH_3_OH and found that the undesirable side reactions could be greatly suppressed [74], which has attracted tremendous attention and become an efficient approach to chemically recycling PET [71]. Recently, McKeown and coworkers successfully achieved the organocatalyzed PET methanolysis with [NMe_4_]^+^[OCO_2_Me]^−^ as catalyst for the first time [23]. The produced DMT was isolated in a moderate yield of 72% under ambient pressure at around 100 °C.

Besides, Ding et al. [75] put forward plastic-catalyst interfacial engineering strategy to achieve the solvent-free depolymerization of PET waste through vapor phase methanolysis at relatively low temperatures (160–180 °C). Trace amounts of ZnO could be deposited on PET surfaces based on electrostatic adsorption, which was able to efficiently catalyze the depolymerization in methanol vapor.

However, the methanolysis products of commercial PET commonly contain some undesirable mixtures such as alcohols, glycols and phthalate derivatives except DMT, the isolation and purification of which will raise the whole cost of methanolysis, thereby unable to compete with inexpensive petro-based counterparts.

#### 3.1.3 PAT glycolysis

PAT glycolysis is a well-established industrial method that has been realized by many large corporations like Shell, DuPont, and Eastman Kodak. This method commonly uses an excess of glycol to undergo transesterification with PATs at very high temperature over 170 °C. An assortment of glycols including EG, PG, BD, diethylene glycol (DEG), et al. have been utilized for PAT glycolysis [14,71]. Among them, EG is the most common degradation agents for PAT glycolysis, leading primarily to BHET and oligomers. The resultant BHET can not only repolymerize to PET but also unsaturated polyester resins.

In 2011, Hedrick et al. used the organocatalyst TBD for the first time to catalyze PET glycolysis [76], affording BHET in a relatively high yield (78%). Later, Sardon and coworkers developed a protic ionic salt (PIS) catalyst preparing by equimolally mixing methane sulfonic acid (MSA) and TBD for PET glycolysis [77,78], which can completely degrade PET within 2 h, attaining pure BHET in a yield of 91%. In virtue of the remarkable thermal stability (resisting degradation > 400 °C) of the TBD:MSA salt, this PIS catalyst is able to be recovered for at least 5 times with no apparent decline in catalytic activity. Remarkably, they also demonstrated the selective and sequential glycolysis of bisphenol A-based PC and PET with TBD:MSA salt as catalyst, due to their similar depolymerization mechanisms but large energetic differences. Furthermore, Saito et al. recently developed another novel PIS-based organocatalysts by mixing TBD and trifluoracetic acid (TFA), in which the TFA anion can provide high conjugate basicity with weaker ionic interaction, thereby able to enhance the reactivity of EG in PET glycolysis [79]. This tailored catalyst design allows for 100% conversion of PET to BHET within 2 h.

Recently, Xiao and Su et al. realized an effective glycolysis of waste PET using cyanamide as catalyst, achieving nearly 100% BHET yield [80]. Experimental and calculation results confirmed that the high catalytic activity of cyanamide is ascribed to the formation of strong hydrogen bonding interactions between cyanamide and PET or EG. The formed hydrogen bonding between PET and cyanamide increased the electrophilicity of PET's C=O groups, while the hydrogen bonding generated between cyanamide and EG enhanced the nucleophilic attack ability of EG's OH groups, thereby accelerating the glycolysis rate of PET.

Besides, a series of neutral, acidic, and basic IL catalysts have also been utilized in the catalysis of PET glycolysis. In 2020, Lu and Zhang et al. demonstrated the catalytic activities of diverse choline-based ILs for PET glycolysis [81]. They found that choline acetate ([Ch][OAc]) can achieve a relatively better catalytic result under mild depolymerization conditions compared with conventional imidazolium metal-based ILs. Soon after, D'Anna et al. reported a kind of sustainable cholinium ILs based on amino acid as catalysts for PET glycolysis [82]. By the optimization of reaction conditions, they discovered that [Ch][Gly] is the best catalyst, which can achieve a good conversion up to 85% and a moderate yield (∼51%) of BHET from a commercial PET bottle in 6 h at 150 °C.

Pickering emulsion interfacial catalysis (PEIC) has been recognized as an efficient catalytic system, which can overcome the problem of low reaction efficiency in heterogeneous reactions caused by the incompatibility of hydrophobic/hydrophilic reactants. Recently, Song and Wu et al. [83] developed a stable hot PEIC system for efficient PET glycolysis by using modified asymmetric nanonets as emulsifier. Due to the large emulsion interface area and catalyst enrichment at the emulsion droplets interface, 100% depolymerization of PET with BHET yield up to 90% was achieved at 170 °C within 5 min. Moreover, this PEIC system still exhibited excellent PET depolymerization rate even after six cycles.

#### 3.1.4 PAT aminolysis and ammonolysis

When aliphatic amines are added, PAT can undergo aminolysis, owing to their stronger nucleophilicity compared with alcohol counterparts. Early in 2006, Shukla and Harad realized the depolymerization of waste PET via aminolysis using glacial acetic acid or sodium acetate and an excess of ethanolamine to afford bis(2-hydroxyethylene) terephthalamide (BHETA) in a high yield up to 91% [84]. Later, the same group developed a novel PET aminolysis strategy by tactfully combining microwave with cheap non-toxic metal salts as catalyst, such as sodium/potassium sulfate, sodium bicarbonate, and sodium acetate, providing excellent yield (> 85%) within minutes [85]. In 2013, Fukushima and Hedrick et al. reported the organic base TBD-catalyzed aminolysis of PET waste [86], using a variety of aromatic, allylic, and aliphatic amines. The obtained crystalline terephthalamides can also be used to prepare high value-added polymers like polyurethanes, poly(ester amide)s, hydrogels, and so on. Besides, researchers also convinced that DES are effective organocatalysts to catalyze the aminolysis of PAT. In 2016, Musale and Shukla reported the first example of DES-catalyzed degradation of waste PET bottle [87], using diethanolamine or ethanolamine. Pure BHETA and *N*^1^,*N*^1^,*N*^4^,*N*^4^-tetrakis(2-hydroxyethyl)-terephthalamide in yields of 95% and 82% respectively, were obtained within 30 min under reflux.

Besides, using liquor ammonia, PET ammonolysis also can be achieved at high temperature (70∼180 °C) under pressure [14], with or without the addition of catalyst, typically Zn(OAc)_2_. The depolymerization products mainly include terephthalamide, 1,4-benzene dicarboxamide, and EG. Compared with other depolymerization methods, PET ammonolysis received less interests, probably owing to its narrow substrate range.

#### 3.1.5 Cyclodepolymerization of PATs

Cyclodepolymerization (CDP) is another important method for PAT recycling, in which the polyesters in dilute solutions can be transformed to the cyclic oligoesters by taking advantage of the ring-chain equilibria, first reported by Brunelle et al. [[88], [89], [90]]. Different solvents and catalysts have been employed for PET cyclodepolymerization at low concentrations. In 1997, Bryant and Semlyen et al. [91] used commercial PET as starting reactants to fabricate cyclic oligo(ethylene terephthalate)s (COETs) with several esterification catalysts under refluxing conditions. They found that Zn(OAc)_2_ is capable of effectively catalyzing CDP of PET, and the larger cyclic oligomers can be generated in a good yield with the increment of the solvent dilution ratio. Later, MacKnight et al. reported two methods to obtain ethylene terephthalate cyclic oligomers either through a direct synthesis approach or CDP of PET waste [92], in a dilute *o*-dichlorobenzene solution using titanate catalyst. Purified COETs could be prepared and was capable of polymerizing at 293 °C toward PET with *M*_n_ over 25,000 after about 15 min using antimony trioxide as catalyst.

At the same year, Burch et al. also synthesized the cyclic oligomers of ethylene isophthalate and ethylene terephthalate with high yield and purity by refluxing the hydrocarbon solutions of their linear polymers or oligomers [93]. An assortment of metal catalysts like zirconium ethoxide, aluminum butoxide, antimony glycoxide, or titanium alkoxides were used for affording cyclic oligomers. The obtained cyclic oligomers can further be polymerized or copolymerized to form high molecular weight PET homo-polyester or its copolyesters (*M*_w_ ∼ 38.9 kg mol^−1^), which commonly are difficult to prepare through conventional polycondensation.

The CDP method can also be used for recycling other semiaromatic polyesters [88], including poly(tetraethylene glycol terephthalate) and poly(decamethylene terephthalate), in chlorobenzene solution under the catalysis of dibutyltin oxide. In 2000, Hodge and coworkers reported the preparation of a variety of cyclic oligo(alkylidene isophthalate)s [94], via dibutyltin oxide catalyzed solution CDP of the corresponding linear polyesters in chlorobenzene or 1,2-dicholorobenzene. In optimal condition, the yield of cyclic oligo(propylene terephthalate)s (COTTs) can achieve 94% [95]. Similarly, cyclic oligo(butylene terephthalate)s (COBTs) can be obtained through dibutyltin oxide catalyzed CDP from commercial PBT in 1,2-dichlorobenzene under the refluxing conditions with yield of 96%, reported by Brittain et al. [96].

Therefore, CDP is also an important chemical recycling method for polyesters. The obtained cyclic oligoesters not only can be used to prepare the original linear polyesters, but also other high value-added polyester-based materials like multiblock copolyesters (mBCPs). Recently, our group reported the synthesis of diverse high molecular weight poly(ether ester) mBCPs [97,98], using different cyclic oligoesters as monomers through a cascade polycondensation-coupling ring-opening polymerization (PROP) method. These mBCPs show excellent mechanical properties, greatly expanding the application scope of cyclic oligoesters.

Some important depolymerization conditions and results of PET are summarized in Table 2. For PET hydrolysis, high yields of TPA could be achieved, whereas, large amount of acid or base catalysts were commonly required, resulting in equipment’ corrosiveness and disposal of large amount of strongly basic or acidic waste water. By contrast, PET alcoholysis or methanolysis could avoid the usage of strong acid or base catalysts and tolerate a wide range of contaminants, but the final products often contained some undesirable mixtures such as alcohols, glycols and phthalate derivatives, thereby raising the whole cost of isolation and purification process. Glycolysis is the simplest and well-established commercial PET recycling method, which has been practiced by some renowned companies, due to its low volatility, relatively mild reaction conditions and continuous production feasibility. However, PET glycolysis often used diverse metal-based organometallic catalysts which may remain in the final products or cause heavy metal pollution, bringing extra environmental concerns. Besides, the recovered products via PET hydrolysis, alcoholysis or glycolysis usually possessed relatively higher prices, hardly competing with their petro-based counterparts. Cyclic oligoesters obtained via CDP technology are of great commercial value due to its high preparation difficulty using common organic synthesis methods. Moreover, cyclic oligoesters can be used to prepare high value-added mBCPs materials. However, the present CDP processes required very low PET concentrations and the usage of large amount of organic solvents, impeding its industrialization.

**Table 2:** **Summary of depolymerization conditions and results of PET**.

Methods	Reagents	Catalysts	Conditions	Products	Yields	Ref.
Hydrolysis	H_2_O	ZSM-5 (50 wt%)	230 °C Microwave	TPA	100%	[66]
Hydrolysis	H_2_O	KOH (140 wt%)	120 °C Microwave	TPA	100%	[67]
Hydrolysis	H_2_O	ZnCl_2_/Zn(OAc)_2_ (<1 wt%)	150–200 °C	TPA	98.7%	[68]
Hydrolysis	H_2_O	Binuclear zinc catalyst (0.2 wt%)	90 °C, pH∼13	TPA	100%	[70]
Alcoholysis	*n*-Butanol	[HO_3_S-(CH_2_)_3—_NEt_3_]Cl-ZnCl_2_ (20 wt%)	205 °C	Dibutyl terephthalate	95.3%	[72]
Alcoholysis	2-Ethyl-1-hexanol	Zn(OAc)_2_/ChCl (5 wt%)	155–185 °C	Dioctyl terephthalate	84.7%	[73]
Methanolysis	CH_3_OH	[NMe_4_]^+^[OCO_2_Me]^−^ (4 wt%)	100 °C	DMT	72%	[23]
Methanolysis	CH_3_OH vapor	ZnO (0.01–1 wt%)	160–180 °C	DMT	100%	[75]
Glycolysis	EG	TBD (0.5–10 mol%)	140–190 °C	BHET	78%	[76]
Glycolysis	EG	TBD:MSA salt (12–50 mol%)	180 °C	BHET	91%	[77]
Glycolysis	EG	TBD:TFA salt (5 mol%)	180 °C	BHET	>96%	[79]
Glycolysis	EG	Cyanamide (2.5–15 wt%)	140–190 °C	BHET	100%	[80]
Glycolysis	EG	[Ch][OAc] (5 wt%)	180 °C	BHET	85.2%	[81]
Glycolysis	EG	[Ch][Gly] (20 mol%)	150 °C	BHET	51%	[82]
Glycolysis	EG	Zn(OAc)_2_ (33 wt%)	170 °C PEIC system	BHET	90%	[83]
Aminolysis	Ethanolamine	Glacial acetic acid, NaOAc, K_2_SO_4_ (0.3–1.5 wt%)	Reflux	BHETA	91.1%	[84]
Aminolysis	Organic amines	TBD (5 mol%)	110–190 °C	Terephthalamides	>63%	[86]
CDP	1-methyl naphthalene	Zn(OAc)_2_ (0.5 wt%)	240 °C	COEAs	30%	[91]
CDP	Dichlorobenzene	Titanate catalyst (3 mol%)	240 °C	COEAs	50%	[92]
CDP	Hydrocarbons	Titanium alkoxides	>200 °C	COEAs	87.5%	[93]

#### 3.1.6 Other upcycling methods of PATs

**Fig. 9:**
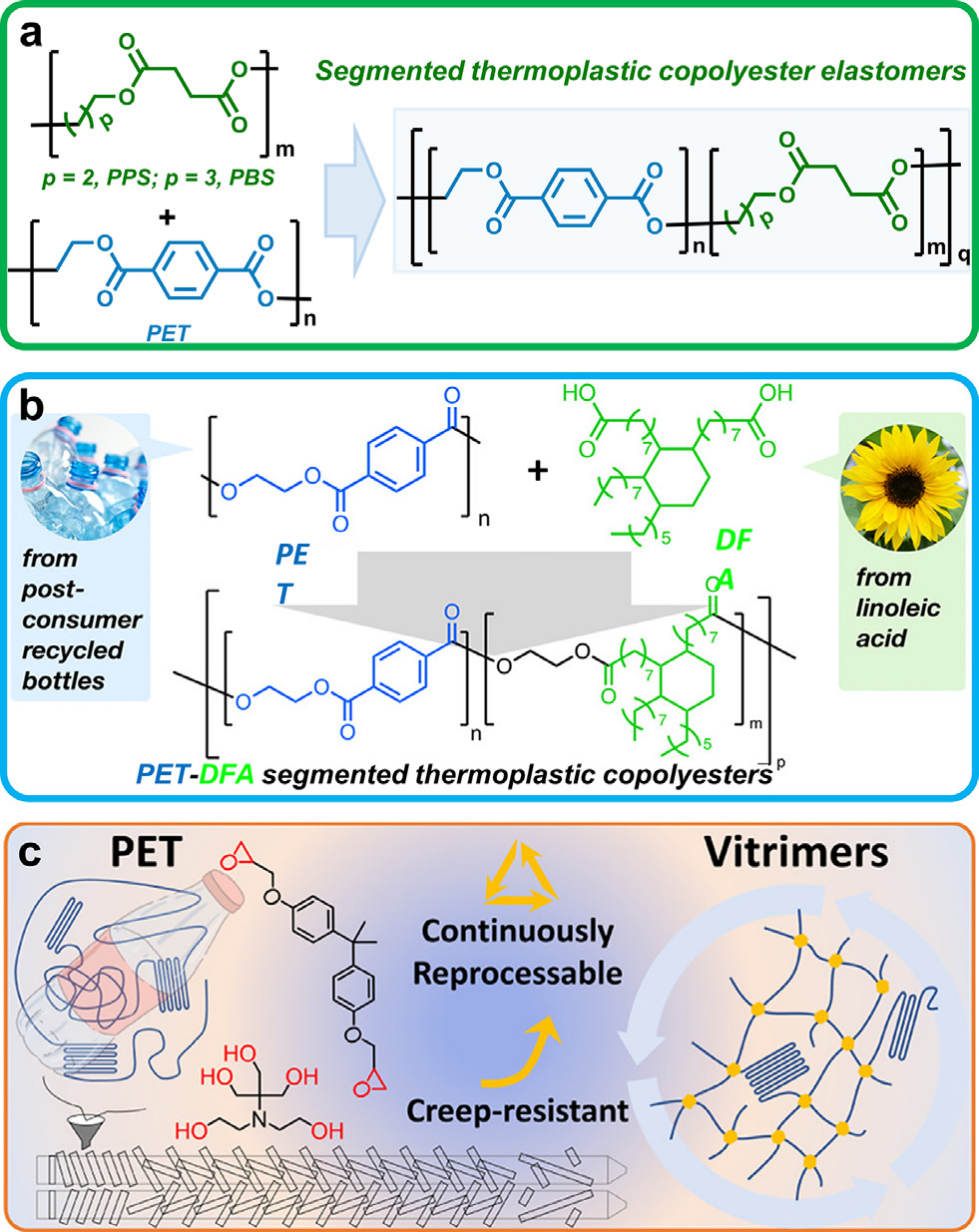
(a) Chemical upcycling of PET through transesterification with PPS or PBS to afford segmented thermoplastic copolyester elastomers. (b) Direct transesterification copolymerization between PET and fatty acid dimer diols in melt state to produce segmented thermoplastic copolyesters. Reproduced from Ref. [100] with permission from American Chemical Society. (c) Reactive reconfiguration of PET with diepoxy and polyol to construct creep-resistant vitrimer networks. Reproduced from Ref. [101] with permission from American Chemical Society.

An appealing chemical recycle method of PATs involves the direct reaction of PATs with a variety of polymers, oligomers, or small molecules to produce high-performance copolyester materials. In 2014, Ghassemi and Schiraldi fabricated a series of novel random copolyesters with tailored mechanical properties [99], based on the transesterification between postconsumer PET and poly(butylene succinate) (PBS) or poly(propylene succinate) (PPS) (Fig. 9a). Recently, Pitet et al. reported a one-pot conversion of postconsumer recycled PET into high-performance random copolyesters [100], by virtue of the transesterification between PET and bio-sourced fatty acid dimer (Fig. 9b). The resultant copolyesters showed good mechanical properties. On the other hand, PET also can be employed as a building block to form thermosets. In 2021, Ma et al. reported the transformation of PET to value-added vitrimers by reacting with diepoxy and polyol in a double-screw extruder (Fig. 9c) [101]. The resulting PET vitrimers displayed improved mechanical performance and good reprocessability no matter by injection, extrusion, or compression molding. In addition, Ok and coworkers recently converted PET waste into porous carbons through chemical and physical activation pathways [102], which showed outstanding CO_2_-capture capacities, thereby providing the opportunity for the realization of carbon neutrality.

### 3.2 Chemical recycling of poly(ethylene furanoate)

Poly(ethylene 2,5-furandicarboxylate) (PEF), the structural analogue of PET, is a promising 100% renewable bio-based plastics, owing to its seductive physical properties. The high *T*_g_ and outstanding gas barrier properties make PEF a promising substitution of PET with potential applications in bottle and film industry. However, commercial PEF is synthesized typically via condensation polymerization, which generally needs the continuous removement of the diol by-products to achieve high molecular weights required for good mechanical performance.

**Fig. 10:**
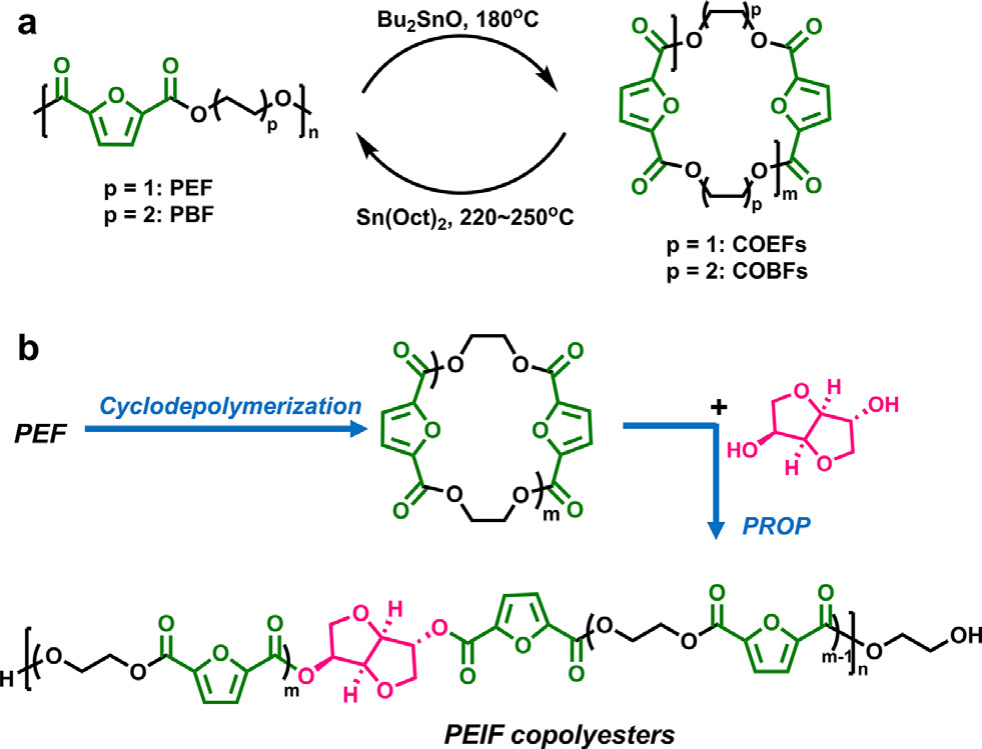
**Cyclodepolymerization and upcycling of PEF**.

In 2016, Muñoz-Guerra et al. reported the CDP method to prepare cyclic oligo(alkylene 2,5-furandicarboxylate)s [103], from corresponding linear PEF and poly(butylene 2,5-furandicarboxylate) (PBF) in the presence of dibutyltin(IV) oxide (Fig. 10a). Both cyclic oligo(ethylene 2,5-furandicarboxylate) (COEFs) and oligo(butylene 2,5-furandicarboxylate) (COBFs) can be obtained in high yields (≥ 72%) in dichlorobenzene at 180 °C. ROP of COEFs and COBFs can afford PEF and PBF with high *M*_w_ above 50 kg mol^−1^, which exhibit the similar thermal properties with those prepared via melt polycondensation. Afterwards, Morbidelli et al. used these COEFs as monomers and bottle-grade PEF with *M*_n_ up to 30 kg mol^−1^can be rapidly synthesized in high conversion over 95% via ROP within minutes [104], suitable for packaging applications.

Very recently, our group reported a novel chemical recycling route of PEF to prepare value-added copolyesters. Biobased high-performance poly(ethylene-*co*-isosorbide furanoate) (PEIF) and PEF-*block*-poly(tetramethylene oxide) mBCPs can be synthesized using COEFs as monomer via the cascade PROP method (Fig. 10b) [105,106]. The obtained PEF-based poly(ether ester) mBCPs show good thermoplastic elastomer properties, while the PEIF fibers prepared by melt spinning exhibit remarkable strength, toughness, and biodegradability.

### 3.3 Chemical recycling of diacid/diol-type aliphatic polyesters

Aliphatic diol/diacid based polyesters, including poly(butylene succinate) (PBS), poly(butylene adipate) (PBA), and poly(ethylene adipate) (PEA), are commonly used in our daily life, synthesized via the condensation polymerization of diols and diacids. They are usually recognized as biodegradable polyesters, and directly thrown away into environment. Thus, exploring an effective chemical recycling strategy to recover these aliphatic polyesters is also meaningful to sustainable development.

**Fig. 11:**
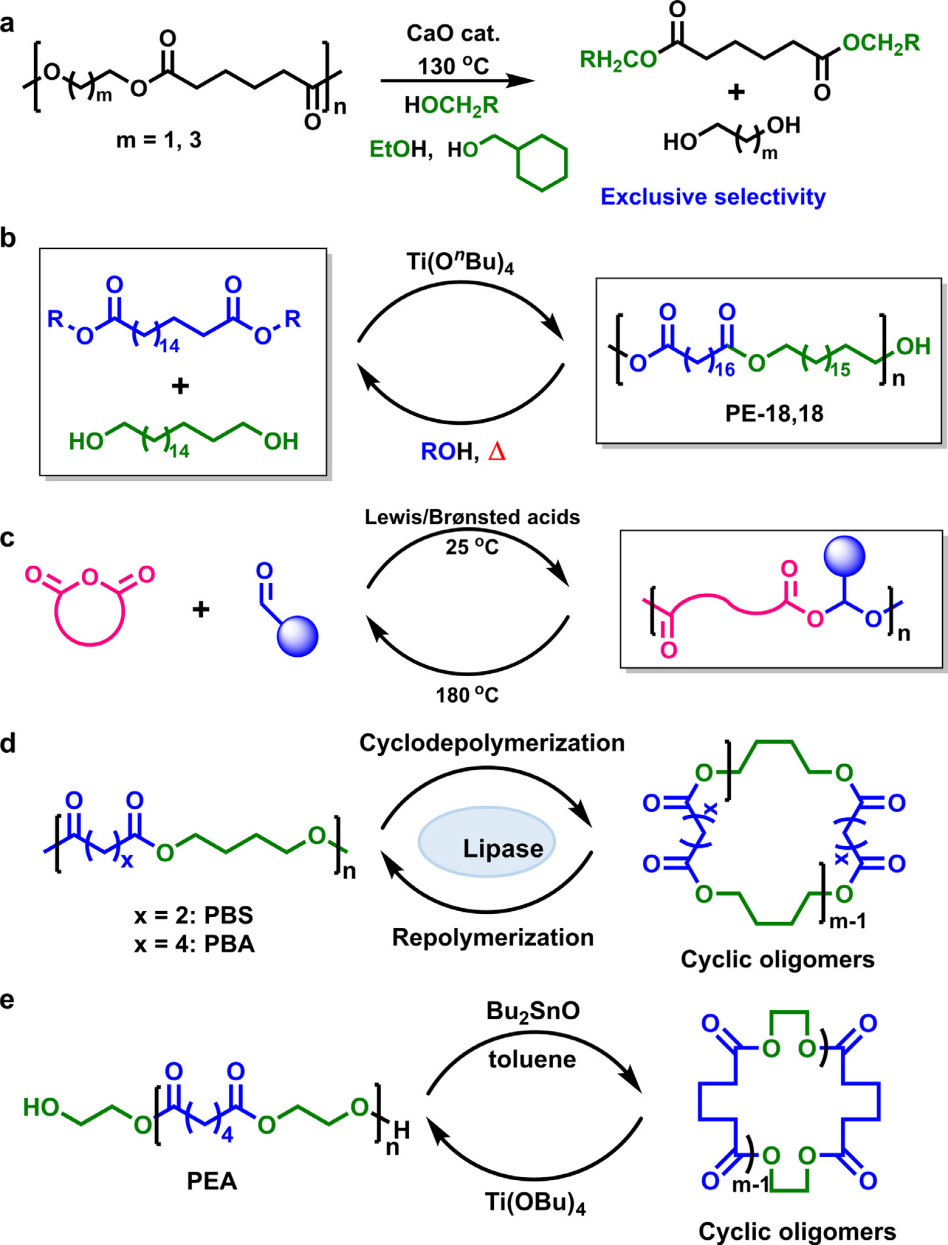
**The polymerization–depolymerization cycle of various diol/diacid-type aliphatic polyesters**.

Diol/diacid-type aliphatic polyesters are able to be chemically recycled by hydrolysis to the diacid and diol precursors at high temperature under the catalysis of alkaline catalysts. Generally, neutralization and purification processes are inevitable, resulting in the consumption of a large quantity of energy. In 2022, Kitiyanan and Nomura et al. [107] demonstrated an effective base-/acid-free depolymerization of aliphatic polyesters, PEA and PBA (Fig. 11a), by using CaO-catalyzed transesterification with cyclohexanemethanol or ethanol to produce corresponding glycol and adipates in high yields (up to 98%). The CaO catalyst displays an excellent reusability. Afterwards, they also reported a highly active transesterification catalyzed by CpTiCl_3_ which enabled a fast complete depolymerization of PEA and PBA at 150 °C [108], affording corresponding monomers exclusively (yield > 99%), thus suited to build up a circular economy.

In 2021, Mecking et al. developed a renewable PE-like polyester material (PE18,18) bearing a small amount of ester bonds as breaking sites (Fig. 11b) [109]. The PE18,18 almost keeps the crystalline structure and mechanical behaviors of high-density PE, and possess excellent chemical recyclability by solvolysis to quantitatively recover the precursors at high temperature. Afterwards, the same group used readily available biobased 1,18-octadecanedicarboxylic acid to polymerize with EG for a novel polyester material, which presented PE-like crystalline structure and mechanical properties [110], and was capable of methanolysis for closed-loop recycling.

Recently, Zhang and coworkers demonstrated a facile and versatile alternating copolymerization of cyclic anhydride and aldehyde to fabricate chemically recoverable polyesters under the catalysis of common Brønsted/Lewis acids following a cationic mechanism (Fig. 11c) [111]. The kinetic and thermodynamic studies evinced that this copolymerization process was chemically reversible, which enabled the resultant polyesters to recover back to the starting monomers at an elevated temperature. Subsequently, the same group further utilized cyclic anhydride and *o*-phthalaldehyde as comonomers to undergo the abovementioned alternating copolymerization for cyclic polyesters [112], which also presented excellent closed-loop recyclability.

CDP is an important method for chemical recycling of aliphatic polyesters. Matsumura's group have pioneered a series of wonderful work in CDP of poly(alkylene alkanedioate)s. In 2003, they successfully applied an efficient lipase-catalyzed CDP to transform aliphatic polyesters, such like PBS, PBA, and poly(butylene adipate-co-succinate) (Fig. 11d) [113], into corresponding repolymerizable cyclic oligomers in acetonitrile or toluene at around 50 °C. The yield of cyclic oligomers can reach 98% at polyester concentration of 2 mg mL^−1^. Interestingly, they also demonstrated that PBA can be completely depolymerized into cyclic oligomers [114], under the supercritical CO_2_ flow at 40 °C within 6 h. Although large quantity of organic solvent and enzyme are required, the immobilized enzyme is capable of repeated usage with no obvious loss in its activity. Furthermore, the resultant cyclic oligoesters are able to repolymerize in toluene using the same lipase to fabricate the parent polyesters with a high *M*_w_ ∼ 98 kg mol^−1^, demonstrating a cyclic polymer economy. Afterwards, they expanded this enzymatic CDP method to other aliphatic polyesters including PLA [115] and PCL [116]*.* Notably, for poly(ester-urethane)s (PEUs) containing ester linkages along the polymer chain [117], they also exhibit excellent lipase-catalyzed recyclability into cyclic ester-urethane oligomers, which can be repolymerized to afford the pristine PEUs with a high molecular weight.

PEA is known as a type of important crystalline aliphatic polyesters, which has been widely applied as plasticizer or macromonomer for the synthesis of various polyurethane materials. Recently, our group demonstrated that under the catalysis of tin catalyst at a mild depolymerization condition, PEA is able to be recycled to a series of cyclic oligoesters, cyclic oligo(ethylene adipate)s (COEAs) with different ring sizes (Fig. 11e) [118]. The attained COEAs can be readily repolymerized to pristine PEA, successfully endowing PEA with a good closed-loop recyclability.

## 4 Conclusions and outlook

In recent years, the chemical recycling of polyesters has drawn enormous attention as a promising new approach to solving the growing polymer pollution problem. The recovered monomers by chemical recycling methods are able to undergo ROP or polycondensation for the pristine polymers showing almost the same material properties with that of virgin polymers, thereby reaching a sustainable cyclic economy. Nevertheless, most of the reported research works are still in the lab stage with significant challenges remaining. In our opinion, there exists the following challenges:

(1) The chemical recycling processes commonly face the urgent problems of high energy consumption and high cost. During the recycling process, the large amount use of organic solvents, strong acids/bases brings about the critical requirement for complex separation and purification procedures, which not only bring about extra environmental issues but tremendously increase the cost of recyclable products. Thus the price can hardly compete with their petroleum-based counterparts. For large-scale industrialization, exploiting a green, efficient and low-cost chemical recycling route of diverse polyesters is a significant direction in the future*.* Especially for commercial poly(alkylene terephthalate)s, a low-cost and economically feasible recycling strategy is incredibly important.

(2) Catalysts is the key for the industrialization of chemical recycling of polyesters. As the depolymerization usually occurs at high temperatures, the coexisted numerous side reactions greatly increase the purification costs. In addition, for postconsumer polymers, the contamination of plasticizers, dirt, water, fillers, pigments, and other polymers, greatly affects the catalyst activity and the efficiency of depolymerization process. Therefore, it is extremely important to develop high efficient, high tolerant depolymerization catalysts to minimize the costs during chemical recycling processes.

(3) Though some new close-looped chemical recycling processes are reported with elegant design, these polyesters typically encounter the problem of higher price with weaker properties that is difficult to be used in our society. It's important to find a chemical recyclable polymer with properties similar to general or engineering plastics. In addition, the processing properties, which are important for large quality production, are usually ignored currently. One of the resolution is the biobased monomers/polyesters with price economically competitive with existing commodity plastics. Efforts are encouraged to be devoted to synthesizing monomers by using sustainable biobased feedstock.

Overall, the chemical recycling of polyesters is still in the infancy stage. There is a very long way from beginning to final scale-up industrialization for this technique compared to these commodity plastics. With the participation of more researchers into this field, we believe these issues will be addressed one by one in the future and chemically recyclable polyesters will make a tremendous influence on the materials science field and sustainable circular economy.

## Declaration of competing interest

The authors declare that they have no conflicts of interest in this work.
